# Digital Health for Australia: Bridging the Rural, Regional, and Remote Health Gap

**DOI:** 10.2196/67460

**Published:** 2025-12-02

**Authors:** Shakeel Mahmood, M Mamun Huda, Kedir Yimam Ahmed, Thapa Subash, Feleke Hailemichael Astawesegn, Anayochukwu Edward Anyasodor, Mohammad Ali Moni, Muhammad J A Shiddiky, Utpal K Mondal, Setognal Birara Aychiluhm, Santosh Giri, Allen Ross

**Affiliations:** 1 Rural Health Research Institute Charles Sturt University Orange Australia

**Keywords:** rural health inequities, digital health adoption, telehealth services, chronic disease management, socioeconomic disadvantage, health workforce, shortages digital literacy gaps, equitable access to care

## Abstract

In rural Australia, recent trends reveal an exponential increase in the rates of physical inactivity, central obesity, metabolic syndrome, and cancer in the population. The limited rural health workforce, which is struggling to meet this growing burden, is boosted by digital technologies such as My Health Record, Cardihab, Healthdirect, and MindSpot, all of which offer opportunities for improved diagnostics, monitoring, and management of chronic diseases. However, implementing proven digital health technologies in rural communities has been challenging on numerous fronts. This perspective aims to (1) highlight the rural health gap and propose a way forward in implementing evidence-based digital health technologies in the rural, regional, and remote communities of Australia and (2) guide future rural health policy.

## Rural Health Gap

**Figure 1:**
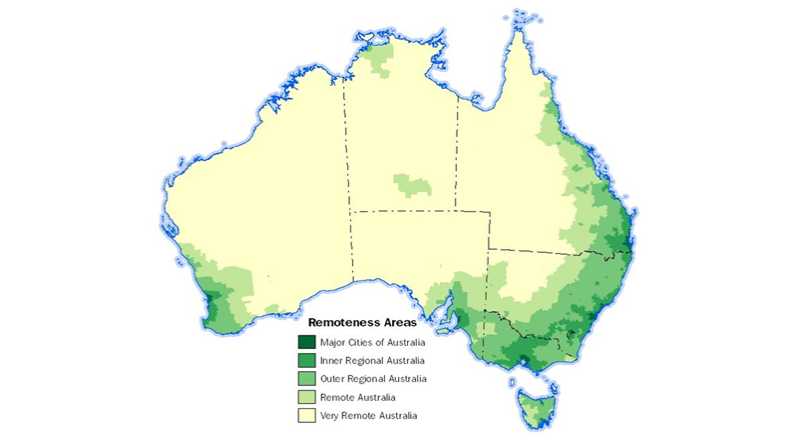
Australian map by remoteness. Adapted from [3].

Approximately 7 million Australians (28%) and 260,000 (32%) First Nations peoples live in rural, regional, and remote (RRR) areas [1,2] (Figure 1 [3]). These regions encompass more than 1700 small towns with populations under 10,000 people [2]. The leading causes of the rural burden of disease are central obesity, type 2 diabetes, coronary heart disease, suicide and self-harm, chronic obstructive pulmonary disease, and lung cancer [4]. Cardiovascular disease is a significant health problem in RRR regions in Australia, where prevalence rates are approximately 20% to 30% higher than in urban areas [4]. Other significant health challenges across RRR communities include chronic kidney disease and dementia and other mental health disorders [5]. As a result, individuals residing in RRR regions experience an age-adjusted mortality rate that is 2.3 times higher than that of those living in major cities [6]. This viewpoint aims to guide policymakers on how to leverage digital health, using global evidence and strategies, to reduce RRR region health inequities.

Several social determinants of health contribute to health disparities in RRR areas, including chronic unemployment, lower educational attainment, historical social injustices, and limited access to fresh fruit and vegetables [7]. For instance, the year 12 completion rate is the lowest in very remote areas compared with that of major cities (55.5% vs 79.4%) [1,8]. Similarly, the median weekly income declines with remoteness, from AUD $1915.2 (the conversion rate at the time of the study was AUD $1=US $0.65) in major cities to AUD $1362.7 in very remote areas [1,8]. The availability of health care professionals also varies substantially, with very remote areas having approximately 205 full-time equivalent doctors per 100,000 people, compared with 427 full-time equivalent doctors per 100,000 in major cities (Table 1) [1,8]. Medicare data from 2022 to 2023 show that general practitioner (GP) visits were lowest in remote and very remote communities, while hospitalization rates (eg, for end-stage renal disease) were almost twice as high in very remote areas and 1.3 times higher in remote areas, with no improvement since 2013 to 2014 [9].

**Table 1:** Sociodemographic and health workforce profile of Australia by remoteness [1,8].

Variables	Major cities	Inner regional	Outer regional	Remote	Very remote
Population size (%)	72	18	8.1	1.2	0.8
Year 12 completion (%)	79.4	74.5	67.6	69.2	55.5
Median weekly household income (AUD $)	1915.2	1637.6	1391.0	1478.7	1362.7
Health care workforce (medical practitioners), FTE^a^ per 100,000	427	311	285	285	205

^a^FTE: full-time equivalent.

## Evidence-Based Digital Health Technology

**Figure 2:**
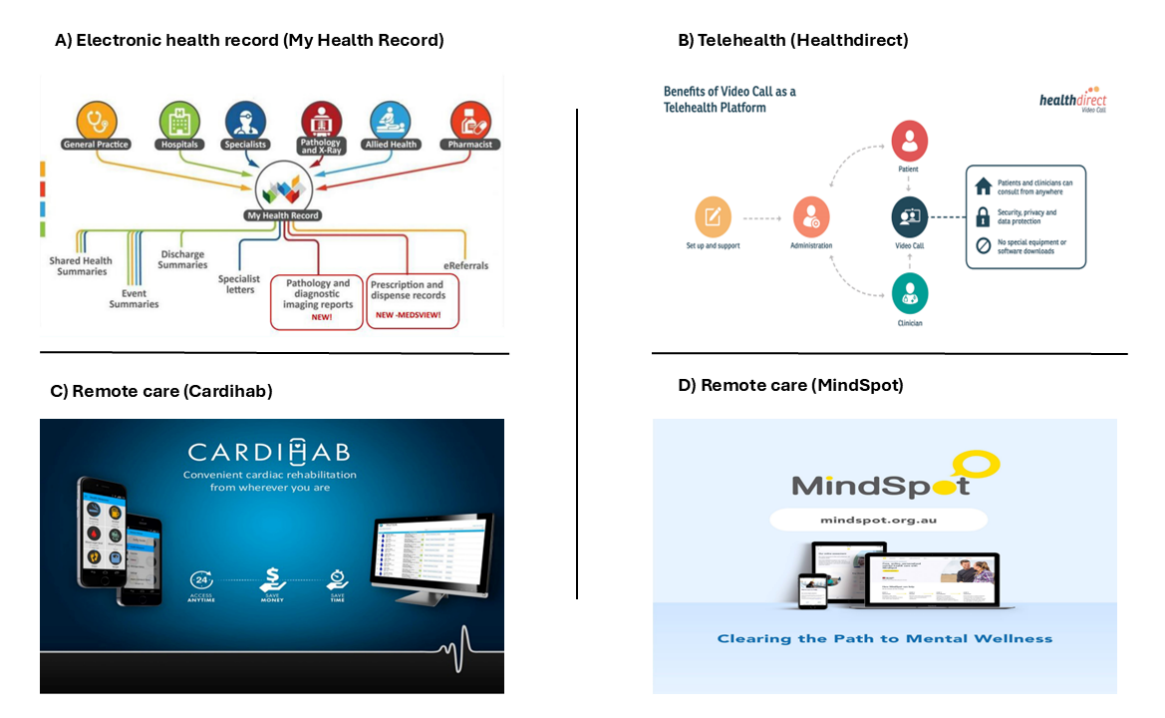
Four available digital platforms in Australia: A) My Health Record securely stores key health information, accessible anytime by patients and healthcare providers, including during emergencies; B) healthdirct operates a ‘virtual front door’ service. It triages patients, provides advice on how they can manage their condition on their own (‘self-care’), and connects them to health services; C) Cardihab is a digital-based cardiac rehabilitation service for patients recovering from a cardiac event or procedure; D) MindSpot is free, confidential psychological assessments and treatments, and access to qualified therapists via online or telephone.

Health care is increasingly being delivered in a technology-driven environment [10]. In Australia, digital health technologies are transforming health care delivery, with significant advancements in electronic medical records (EMRs), telehealth, and remote patient monitoring [11]. EMRs have been widely adopted within GP practices, hospitals, and other service providers, facilitating seamless transfer of patient records among health care providers. Studies indicate that EMR adoption rates in RRR areas often surpass those in urban settings. For example, a 2012 study found that 56% of RRR region practices had adopted EMRs compared with 49% in urban areas [12]. Similarly, another study reported that solo practices in RRR regions were more likely to adopt EMRs than their urban counterparts (41% vs 33%) [13]. My Health Record, a Medicare-funded national digital system, improves care continuity by providing comprehensive, accessible patient information across health care settings (Figure 2) [14].

Telehealth services have also grown substantially, particularly with Healthdirect, a national service funded by federal, state, and territory governments [11]. Healthdirect operates as a “virtual front door,” triaging patients, providing self-care advice, and connecting them to necessary health services. Several regions, including Victoria’s Northern Health, have implemented “Virtual Emergency Departments” using telehealth to triage patients efficiently [15]. The use of telehealth has extended to innovative outpatient care models, such as the Royal Prince Alfred Hospital’s “Spirituals.” In the Aboriginal Community Controlled Health Organisation sector, telehealth supports culturally appropriate health care through methods like “Store-and-Forward.” Specialized imaging captures patient data and forwards them to specialists for diagnosis and treatment, supporting complex conditions like heart, eye, ear, and diabetes care [16].

Remote care technologies such as remote patient monitoring and digital therapeutics help in effectively managing chronic conditions and reducing hospital admissions in RRR settings [11]. For example, Cardihab delivers personalized digital cardiac rehabilitation, enabling clinicians to monitor patient progress through an online portal [17]. Mental health digital therapeutics, provided by services like Mental Health Online, MindSpot, and THIS WAY UP offer stand-alone or blended care options [18]. Blended care enhances flexibility, supports RRR access, and integrates digital tools into traditional pathways for cardiac and mental health services [19]. Hospitals also leverage remote patient monitoring, with the Royal Prince Alfred Hospital’s “Virtual Hospital” providing 24/7 multidisciplinary care [20]. The Virtual Clinical Care program in South Australia is another example, aimed at reducing preventable hospital admissions through early intervention and continuous monitoring [21].

Artificial intelligence (AI) integration in health care is still limited, mostly restricted to research settings rather than widespread clinical use [22]. The health sector is one of the least mature industries in terms of AI implementation both in Australia and internationally [23,24]. AI analyzes data to support decisions; telehealth delivers care remotely. Together, they enhance access, efficiency, and personalization [25].

## Implementing My Health Record, Healthdirect, Cardihab, and MindSpot

In 2022, nearly half of RRR Australian regions experienced significant broadband or mobile internet connectivity gaps [6]. RRR Australians face ongoing challenges with internet connectivity, especially in terms of video connectivity, and limited digital literacy, despite the narrowing of the urban-rural digital access gap [26]. Implementing My Health Record, an electronic health record (EHR) system, has been challenging on many fronts (Table 2). Issues relate to usability, safety, and the burden it places on physicians [27]. Other challenges of EHR implementation include data entry issues, inadequate alerting systems, and problems with interoperability [27]. Other concerns include high implementation costs, EHR standardization issues, resistance to change, and initial system use reducing productivity [28]. One survey found that most people (71.2%) knew little about My Health Record, and 29.4% had opted out of the system [29]. My Health Record improved access to information, but health literacy gaps, language barriers, and medical jargon caused misinterpretation and sharing issues. Studies need to assess whether the My Health Record improved Australia’s health care as per the objectives of the 2012 Act [30].

**Table 2:** Resources required for the 4 digital health platforms in Australia [17,31-33].

Resources required		My Health Record (electronic health record)	Healthdirect (telehealth)	Cardihab (remote care)	MindSpot (remote care)
Internet		✓	✓	✓	✓
Broadband connections		✓	✓	✓	✓
Electricity		✓	✓	✓	✓
**Cellphone**					
	Normal phone		✓		✓
	Smart phone	✓	✓	✓	✓
Computer or tablet		✓	✓	✓	✓
**Trained staff**		✓	✓	✓	✓
	IT staff	✓	✓	✓	✓
	Health staff	✓	✓	✓	✓

Healthdirect Australia offers nurse-led telephone triage, crucially supporting RRR areas facing GP shortages and limited health care access*.* The service helps patients determine the most appropriate level of care, reducing unnecessary emergency department visits [34]. Healthdirect specializes in providing virtual health information, advice, and referral. However, the implementation of such digital health services can be challenging. A key barrier to implementing is the complexity of navigating Australia’s health care system [35], where coordination between primary and specialist care is poor, especially affecting access in underserved and RRR communities, according to the Grattan Institute report [36]. This leads to missed follow-ups and fragmented care, especially for patients with chronic or complex conditions [36]. Failure to involve a patient’s GP in all aspects of patient care can disrupt continuity of care and compromise the provision of high-quality, cost-effective care [37], particularly in Australia, where the health care system heavily relies on GPs for primary care. A theoretically based implementation plan that can be reported transparently is another pressing requirement [38]. Furthermore, allocating resources to implement the intervention within routine care can also pose difficulties [38].

Cardihab offers digital cardiac rehabilitation [38], improving access and participation in RRR areas, where travel distances historically limited program engagement. In North Queensland, inadequate discharge planning and referrals limit cardiac rehabilitation access for hospital-treated patients [39]. Cost-effectiveness shows savings via fewer rehospitalizations, but sustainability requires continuous Medicare funding and digital skills training for clinicians and patients [40,41]. Despite these benefits, integrating digital cardiac rehabilitation into routine health care remains challenging. Service underuse persists due to a combination of patient and provider factors. Barriers include high costs, staff resistance, training demands, usability issues, data privacy, technical limitations, migration difficulties, and interoperability challenges [38]. National data show that among the 49,900 eligible patients between 2013 and 2015, only 30% were referred to cardiac rehabilitation, and of those referred, fewer than one-third (28%) attended the program [42].

MindSpot delivers online therapy to underserved RRR Australians [43], effectively reducing depression and anxiety symptoms comparable to face-to-face psychological care [44]. Despite these benefits, uptake remains disproportionately low; only 18.9% of registrants are from RRR areas, where approximately 29% of Australians live [45]. Barriers include persistent digital access issues, cultural acceptability concerns, and limited targeted promotion in Indigenous and farming communities [46]. Furthermore, evidence is lacking about the effectiveness of digitally delivered mental health care in real-world settings [45]. Challenges related to the collection of outcome data also persist [47], highlighting the complexities of implementing digital health services in RRR settings.

## Additional Examples of Telehealth and AI in RRR Settings

Telehealth is also being leveraged beyond these 4 platforms. For example, the Royal Flying Doctor Service delivers telehealth consultations to remote patients for chronic disease management and mental health, reducing emergency retrievals [48]. The Northern Territory Health Department outlines a structured approach to early detection and management of type 2 diabetes in Aboriginal youth [49]. This includes referral pathways and screening protocols that align with telehealth-enabled services, such as diabetic obesity screening [49]. In addition, Healthy Rural Hearts telehealth in NSW delivers personalized nutrition therapy for cardiovascular disease risk, although its effectiveness and patient engagement are still under evaluation [50].

A systematic review finds evidence of the promising potential of portable ultrasound in RRR Australia, enabling nurses to capture scans and receive automated reports for obstetric and cardiac diagnostics [51]. For example, studies in remote Indigenous communities have shown feasibility and improved diagnostic capacity when nurses use portable ultrasound devices under tele-supervision from specialists [51]. This approach reduces the need for patient travel, supports timely clinical decision-making, and strengthens local health care capacity in under resourced areas. AI-supported triage tools in Healthdirect call systems also show promise, as evaluated in a 2025 Macquarie University usability study of the Triage Platform [52]. The platform can aid in detecting high-acuity cases but is still in the pilot stages, needing further validation, especially in RRR areas [52].

## Current Utilization

Australian residents who are registered with Medicare or an individual health care identifier can access My Health Record via their mobile app or via the official webpage [53,54]. Mental Health Online provides tailored cognitive behavioral therapy programs for anxiety, depression, posttraumatic stress disorder, and obsessive-compulsive disorder, guided by its Electronic Psychological Assessment Screening System [55]. My Health Record is particularly valuable for continuity of care when patients need to travel across large distances for specialist consultations. Rural GPs report improved care coordination, especially for patients with complex multimorbidities, where local facilities cannot provide specialist services [36]. Rural solo practices adopt centralized health records more than metropolitan groups owing to benefits in sparse referral networks [34]. However, full integration is limited because of persistent opt-out rates in remote areas, partly due to mistrust and low awareness [11]. Healthdirect offers 24/7 helplines and online resources, providing Australians with accessible, trusted, and quality health information and advice [54,56]. Cardihab has been used by Australian private health insurers, clinicians, and patients in Queensland, Victoria, and South Australia [57]. Cardihab, a class I medical device registered in Australia, is being implemented nationwide to support cardiac rehabilitation services [58]. In 2023, 27% of MindSpot users were from RRR areas [59]. Mindspot provides mental health services to underserved populations, including Indigenous Australians, those facing access barriers, and residents outside major cities [60]. THIS WAY UP offers evidence-based online mental health courses, supporting RRR region practitioners and providing culturally safe tools for Indigenous communities via the WellMob portal [61].

## International Case Studies

In Canada, the Ontario Virtual Care Program, initiated in 2006 and supported by the Ontario Telemedicine Network, introduced virtual visits to assist patients in remote locations [62]. This initiative aimed to enhance access to care and improve continuity and coordination of services. The program has evolved to integrate virtual care services within the health system, benefiting underserved populations, including those in RRR communities [63]. Additionally, the Ontario Virtual Care Program enhances sustainability by enabling policymakers to iteratively adapt service delivery models to RRR realities [64].

In Finland, the OuluHealth ecosystem in northern Finland brings together stakeholders from the health sector to facilitate cooperation and improve health and social care services. Collaboration in medicine, wireless technology, ecosystem enabling, and big data facilitates rapid responses to digital health challenges, effectively addressing societal needs [65]. Integrated digital health models with robust infrastructure can improve health care access and continuity in remote regions.

The Framework for Digital Health Equity study [66] emphasizes that equitable digital health solutions must address barriers for marginalized, Indigenous, and older adults in rural US communities. Ensuring these groups have access to technology and the necessary digital skills will be crucial in reducing health care disparities and promoting digital health equity.

## Evaluation Approaches

The evaluation of digital health programs in RRR regions relied on a mixed methods design that incorporates the following:

- Service use data (eg, Medicare claims or platform analytics) to quantify reach and uptake [67]
- Health outcomes (eg, hospital admission rates or disease-specific metrics such as HbA_1c_ also known as glycated hemoglobin levels for diabetes) [68,69]
- Economic evaluations assessing cost-effectiveness considering patient travel, workforce efficiency, and avoided hospitalizations [40]
- Qualitative feedback from patients, clinicians, and community leaders in RRR areas to assess cultural safety, usability [70], and trust

## Way Forward

**Figure 3:**
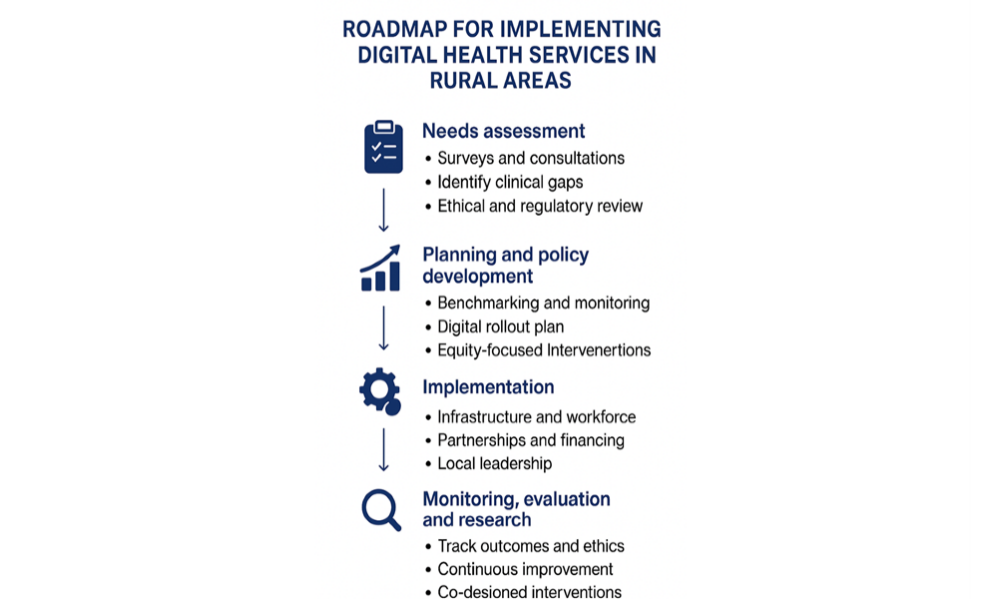
The key implementation steps.

Implementing digital health services in RRR areas is a complex task requiring careful planning and strategic investment (Figure 3 [15,53,57,71,72]) [26]. Rural communities’ needs must be assessed to identify current and future essential clinical services [41,73]. This will involve a cross-sectional survey, community consultation, and data analysis [41,73]. Analyzing ethical risks, privacy, security, consent, and regulations can enhance telehealth governance and strengthen digital health policy recommendations [11,74]. Furthermore, systematic data collection would enable benchmarking, monitoring, and ethical oversight, driving continuous improvement across digital health services [75]. Federal and state health authorities must prioritize RRR region–specific data and programs to address unique needs [2]. Once the needs and current capacity are identified, a plan to roll out digital services needs to be developed to complement the existing clinical practice. One such project was the implementation of electronic prescribing infrastructure in 2021, where 36% of GPs generated e-prescriptions, and 95% of the Pharmaceuticals Benefits Scheme pharmacies dispensed them [11]. RRR health service implementation is expensive because of the costs incurred through infrastructure, equipment, skilled workforce, tailored interventions, multilevel strategies, local leadership, and advocacy needs [76,77]. Socioeconomic, connectivity, literacy, and funding gaps worsen RRR region digital health disparities, necessitating inclusive, equity-focused strategies [78-80]. Government subsidies for devices and training programs for RRR health care workers can help reduce barriers and strengthen digital health adoption [81,82]. Government funding; partnerships (policymakers, health care providers, and technology developers) with the private sector; and innovative financing models should be explored to cover these costs [76]. Effective implementation of digital health in RRR areas requires long-term Medicare funding, strengthened public-private partnerships, and targeted workforce training. Equitable policies, reliable infrastructure, and practical integration of telehealth, EHRs, and remote monitoring are essential. Overcoming RRR region digital health barriers in Australia requires community engagement, device subsidies, digital literacy, and socioeconomic support for sustainable, accessible, and culturally appropriate care. Coordinated planning, cost management, and sustainable funding enhance efficiency, scalability, and long-term impact of digital health services in underserved RRR communities. Future research should co-design dietary interventions combining community-based support with digital approaches for greater effectiveness [50]. By aligning digital health initiatives with the realities of RRR region practice, Australia can move closer to bridging the health gap and ensuring equitable access to care for all communities.

## Abbreviations

EHR: electronic health record
EMR: electronic medical record
GP: general practitioner
RRR: rural, regional, and remote
