# Correction: Digital Health for Australia: Bridging the Rural, Regional, and Remote Health Gap

**DOI:** 10.2196/89755

**Published:** 2025-12-29

**Authors:** Shakeel Mahmood, M Mamun Huda, Kedir Yimam Ahmed, Thapa Subash, Feleke Hailemichael Astawesegn, Anayochukwu Edward Anyasodor, Mohammad Ali Moni, Muhammad J A Shiddiky, Utpal K Mondal, Setognal Birara Aychiluhm, Santosh Giri, Allen G Ross

**Affiliations:** 1 Rural Health Research Institute Charles Sturt University Orange Australia

In “Digital Health for Australia: Bridging the Rural, Regional, and Remote Health Gap” [1], the authors noted one error.

The name of the final author in the authorship list originally read as follows:

Allen Ross

The final author’s name now reads:

Allen G Ross

The correction will appear in the online version of the paper on the JMIR Publications website, together with the publication of this correction notice. Because this was made after submission to PubMed, PubMed Central, and other full-text repositories, the corrected article has also been resubmitted to those repositories.
